# Implementation of Rotavirus Surveillance and Vaccine Introduction — World Health Organization African Region, 2007–2016

**DOI:** 10.15585/mmwr.mm6643a7

**Published:** 2017-11-03

**Authors:** Jason M. Mwenda, Rachel M. Burke, Keith Shaba, Richard Mihigo, Mable Carole Tevi-Benissan, Mutale Mumba, Joseph Nsiari-Muzeyi Biey, Dah Cheikh, Alain Poy, MSc, Felicitas R. Zawaira, Negar Aliabadi, Jacqueline E. Tate, Terri Hyde, Adam L. Cohen, Umesh D. Parashar

**Affiliations:** ^1^World Health Organization Regional Office for Africa, Brazzaville, Republic of the Congo; ^2^Division of Viral Diseases, National Center for Immunization and Respiratory Diseases, CDC; ^3^Epidemic Intelligence Service, CDC; ^4^Department of Immunization, Vaccines, and Biologicals, World Health Organization, Geneva, Switzerland; ^5^Global Immunization Division, Center for Global Health, CDC.

Rotavirus is a leading cause of severe pediatric diarrhea globally, estimated to have caused 120,000 deaths among children aged <5 years in sub-Saharan Africa in 2013 (*1*). In 2009, the World Health Organization (WHO) recommended rotavirus vaccination for all infants worldwide (*2*). Two rotavirus vaccines are currently licensed globally: the monovalent Rotarix vaccine (RV1, GlaxoSmithKline; 2-dose series) and the pentavalent RotaTeq vaccine (RV5, Merck; 3-dose series). This report describes progress of rotavirus vaccine introduction (*3*), coverage (using estimates from WHO and the United Nations Children’s Fund [UNICEF]) (*4*), and impact on pediatric diarrhea hospitalizations in the WHO African Region. By December 2016, 31 (66%) of 47 countries in the WHO African Region had introduced rotavirus vaccine, including 26 that introduced RV1 and five that introduced RV5. Among these countries, rotavirus vaccination coverage (completed series) was 77%, according to WHO/UNICEF population-weighted estimates. In 12 countries with surveillance data available before and after vaccine introduction, the proportion of pediatric diarrhea hospitalizations that were rotavirus-positive declined 33%, from 39% preintroduction to 26% following rotavirus vaccine introduction. These results support introduction of rotavirus vaccine in the remaining countries in the region and continuation of rotavirus surveillance to monitor impact.

The status of rotavirus vaccine introduction and 2016 WHO/UNICEF estimates of national vaccination coverage were obtained from the WHO repository (*3*,*4*). Among African Region countries that have introduced rotavirus vaccine into their national Expanded Programs on Immunization, most recommend that rotavirus doses coincide with administration of the infant doses of diphtheria and tetanus toxoids and pertussis (DTP) vaccine (at ages 6 and 10 weeks for RV1 and at ages 6, 10, and 14 weeks for RV5); most countries are using RV1 (*5*). Because the WHO/UNICEF estimates do not include a coverage estimate for the first dose of rotavirus vaccine or the second dose of DTP vaccine, rotavirus vaccination coverage (completed series of either 2 RV1 or 3 RV5 doses) was compared with first-dose and third-dose coverage for DTP. Countries that have introduced rotavirus vaccine were grouped by year of vaccine introduction for analysis.

Rotavirus surveillance data were collected through sentinel hospitals participating in the African Rotavirus Surveillance Network (ARSN), which was established in four countries in 2006 and had expanded to 29 countries by 2016 (Figure) (*6*). Surveillance staff members at sentinel sites in ARSN enroll children aged <5 years who are hospitalized for acute diarrhea (≥3 looser than normal stools in a 24-hour period before hospitalization, with duration of illness ≤7 days before hospitalization) and collect a stool specimen, which is tested for rotavirus using an enzyme immunoassay. Countries were included in this analysis if their sites collected and tested at least 80 specimens over at least 11 months in a given year. The percentage of tested specimens that were positive for rotavirus was calculated in the vaccine preintroduction and postintroduction periods, by country. The year of rotavirus vaccine introduction was considered a transition period and was excluded from calculations.

**FIGURE:**
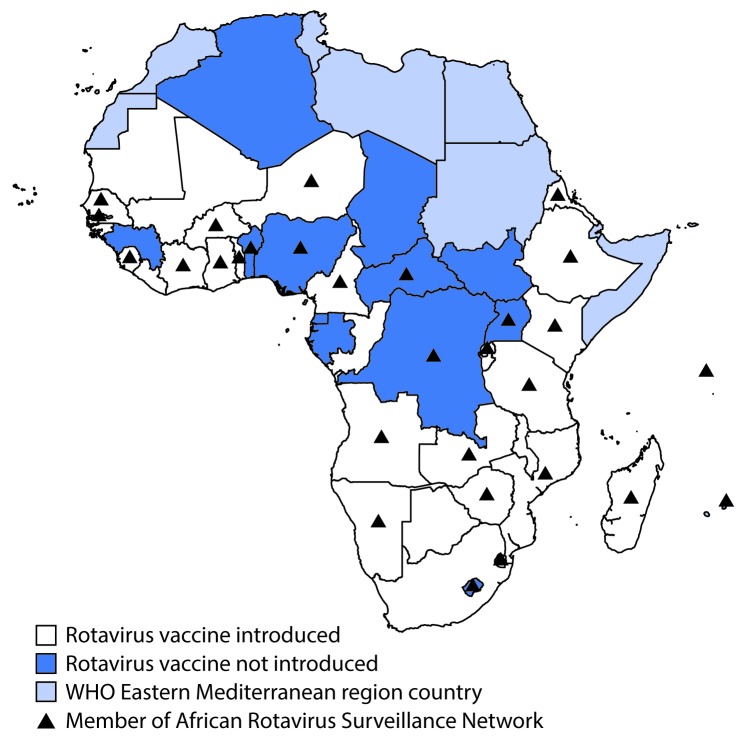
Rotavirus vaccine introduction status — World Health Organization (WHO) African Region, 2016

Overall, 31 (66%) countries in the region had introduced rotavirus vaccine into their national immunization schedules by December 2016, with 26 introducing RV1 and five introducing RV5 (Table 1). Among all countries, completed series rotavirus vaccination coverage was 77% (population-weighted average); national coverage ranged from 24% (Sao Tome and Principe, 2016 introduction) to 98% (Rwanda, 2012 introduction). When grouping by year of vaccine introduction, the highest overall population-weighted coverage (82%) was in countries that introduced the vaccine before 2014. These same countries also had the smallest average percentage-point difference between completed rotavirus vaccination coverage and DTP1 coverage (overall, 11 percentage points less than DTP1).

**TABLE 1:** Percentage coverage with first and third DTP vaccine doses and completed rotavirus (RV) vaccination series — World Health Organization African Region, 2016

Country	Year RV vaccine introduced	RV vaccine type	Coverage (%)			Percentage-point difference in coverage	
			DTP1	DTP3	RV (completed series)	DTP1 versus completed RV	DTP3 versus completed RV
Countries introducing RV vaccine 2009–2013*	**—**	**—**	**93**	**88**	**82**	**11**	**6**
South Africa	2009	RV1	78	66	73	5	−7
Botswana	2012	RV1	98	95	95	3	0
Ghana	2012	RV1	94	93	94	0	−1
Malawi	2012	RV1	89	84	81	8	3
Rwanda^†^	2012	RV5	99	98	98	1	0
Tanzania	2012	RV1	99	97	96	3	1
Burkina Faso	2013	RV5	95	91	91	4	0
Burundi	2013	RV1	97	94	96	1	−2
Ethiopia	2013	RV1	86	77	63	23	14
Gambia^†^	2013	RV5	99	95	95	4	0
Zambia	2013	RV1	99	91	90	9	1
Countries introducing RV vaccine 2014*	**—**	**—**	**89**	**79**	**73**	**16**	**6**
Angola	2014	RV1	79	64	53	26	11
Cameroon	2014	RV1	92	85	80	12	5
Republic of the Congo	2014	RV1	85	80	80	5	0
Eritrea	2014	RV1	97	95	96	1	−1
Kenya	2014	RV1	96	89	74	22	15
Madagascar	2014	RV1	84	77	78	6	−1
Mali	2014	RV5	86	68	60	26	8
Mauritania	2014	RV1	87	73	73	14	0
Namibia	2014	RV1	98	92	86	12	6
Niger	2014	RV1	87	67	61	26	6
Senegal	2014	RV1	96	93	93	3	0
Sierra Leone	2014	RV1	97	84	95	2	−11
Togo	2014	RV1	93	89	90	3	−1
Zimbabwe	2014	RV1	94	90	91	3	−1
Countries introducing RV vaccine 2015–2016*	**—**	**—**	**92**	**81**	**73**	**19**	**8**
Guinea-Bissau	2015	RV1	95	87	61	34	26
Mauritius	2015	RV1	97	96	92	5	4
Mozambique	2015	RV1	90	80	76	14	4
Swaziland	2015	RV1	96	90	95	1	−5
Liberia	2016	RV1	99	79	48	51	31
Sao Tome and Principe	2016	RV5	97	96	24	73	72

**Abbreviations:** DTP = diphtheria and tetanus toxoids and pertussis; DTP1 = first dose of DTP vaccine; DTP3 = third dose of DTP vaccine; RV1 = monovalent RV vaccine (Rotarix); RV5 = pentavalent RV vaccine (RotaTeq).

* Summary data for introduction period are population-weighted averages.

^†^ Country initially introduced RV5, but switched to RV1 in 2017.

Surveillance data were available for 12 and 18 countries during the vaccine preintroduction and postintroduction periods, respectively (Table 2). The average percentage of tested stool specimens that were positive for rotavirus during the vaccine preintroduction period was 41%, ranging from 20% (Ethiopia) to 51% (Togo). During the vaccine postintroduction period, the average percentage of rotavirus-positive specimens was 24%, ranging from 12% (Madagascar) to 41% (Mauritius). In the 12 countries with both vaccine preintroduction and postintroduction data, rotavirus positivity declined by 33% overall (range = 2%–62%), from 39% in the preintroduction period to 26% in the postintroduction period (p<0.001); in these countries, the overall population-weighted 2016 completed rotavirus vaccination series coverage was 82%. In 2016, the overall percentage of positive rotavirus stool specimens was 26% in countries that had introduced the vaccine in 2015 or earlier, and 43% in countries that had not yet introduced the vaccine (p<0.001).

**TABLE 2:** Rotavirus (RV) stool specimen surveillance results, by country and vaccine introduction status — World Health Organization African Region, 2008–2015

Country	Year RV vaccine introduced	Vaccine preintroduction period			Vaccine postintroduction period			% decline in RV positivity*
		Years included	RV specimens tested	No (%) positive	Years included	RV specimens tested	No. (%) positive	
**Countries introducing 2012–2013**	—	—	9,916	3,685 (37)	—	20,389	5,544 (27)	27%
Ghana	2012	2008, 2010–2011	2,374	1,161 (49)	2013–2016	1,494	405 (27)	45%
Rwanda	2012	2011	240	121 (50)	2013–2015	2,237	447 (20)	60%
Tanzania	2012	2009–2010	852	308 (36)	2013–2016	8,615	2,186 (25)	30%
Zambia	2012	2007–2011	4,519	1,700 (38)	2013–2016	5,227	1,700 (33)	14%
Burkina Faso	2013	NS	NS	NS	2014–2016	1,889	615 (33)	NS
Ethiopia	2013	2008–2012	1,931	395 (20)	2014–2016	822	165 (20)	2%
Gambia	2013	NS	NS	NS	2014	105	26 (25)	NS
**Countries introducing 2014**	—	—	14,062	5,628 (40)	—	6,704	1,552 (23)	42%
Angola	2014	NS	NS	NS	2015	229	41 (18)	NS
Cameroon	2014	2008–2013	3,449	1,398 (41)	2015–2016	973	197 (20)	50%
Kenya	2014	2007–2013	4,406	1546 (35)	2015–2016	688	158 (23)	35%
Madagascar	2014	NS	NS	NS	2015–2016	451	56 (12)	NS
Niger	2014	NS	NS	NS	2016	168	22 (13)	NS
Senegal	2014	2011–2013	374	159 (43)	2015–2016	235	38 (16)	62%
Togo	2014	2008, 2010–2013	1,028	526 (51)	2015–2016	319	119 (37)	27%
Zimbabwe	2014	2008–2009, 2011–2013	4,805	1,999 (42)	2015–2016	3,641	921 (25)	39%
**Countries Introducing 2015**	—	—	1,319	626 (47)	—	1,081	330 (31)	36%
Mauritius	2015	2010–2014	1,203	578 (48)	2016	570	235 (41)	14%
Mozambique	2015	NS	NS	NS	2016	420	68 (16)	NS
Swaziland	2015	2013	116	48 (41)	2016	91	27 (30)	28%

**Abbreviations:** NS = No surveillance data available (surveillance not started or data do not meet analysis criteria); RV = rotavirus vaccine.

* Calculated only for countries with data on rotavirus vaccine preintroduction and postintroduction. Percentage declines might not correspond to preintroduction and postintroduction percentages because of rounding.

## Discussion

Countries in the WHO African Region have made significant progress in the introduction of rotavirus vaccines, with 31 (66%) of 47 member countries having introduced rotavirus vaccine into their national schedules by December 2016. In 2016, the overall completed series rotavirus vaccination coverage in these countries was 77%, which was lower than coverage for DTP1 and DTP3. Some of this difference might be attributable to challenges that are common to new vaccine introduction (e.g., it can take time for all vaccination clinics to have reliable cold chain space and a steady stock of a new vaccine). In addition, challenges specific to the recording and reporting of coverage for new vaccines include mid-year introductions, unavailability of updated data tools, and inadequate orientation of health workers on use of vaccine tally sheets. Another factor specific to rotavirus is the issue of age restrictions. Because of concerns about a potential increased risk for intussusception in older infants who receive the vaccine, WHO initially recommended that rotavirus vaccination be administered only to children aged ≤32 weeks (*2*). In 2013, WHO recommended lifting these restrictions based on new data and a risk-benefit analysis (*7*); however, some countries, or some health workers, might still be administering rotavirus vaccine with age restrictions. Additional research is needed to better understand the impact of lifting age restrictions on coverage, and the difference between rotavirus and DTP vaccination coverage.

Surveillance data from ARSN indicate that, among countries with data available both preceding and following rotavirus vaccine introduction, the proportion of rotavirus-positive hospitalizations for diarrhea among children aged <5 years declined 33% following rotavirus vaccine introduction; overall declines were especially notable in countries that had introduced rotavirus vaccine before 2015. These results are particularly encouraging given rotavirus vaccines’ lower efficacy in low-income settings (50%–64% efficacy) than in high-income and middle-income settings (85%–100% efficacy) (*8*), which had raised concerns about the public health impact of their introduction. However, consistent with recent global analyses demonstrating substantial rotavirus vaccine impact across country income strata (*9*), the present analysis suggests that rotavirus vaccination has had a meaningful impact on rotavirus disease in Africa.

Sixteen countries in the WHO African Region had not yet introduced rotavirus vaccine as of December 2016; 10 are eligible for Gavi financial support, four of which have received approval. Apart from funding, other factors can affect rotavirus vaccine introduction and subsequent coverage. Coverage with routinely recommended vaccines, as a marker of immunization system function, highlights several countries in the region where the immunization infrastructure needs strengthening. Armed conflict and natural disasters, experienced by several countries in the region, can further stress immunization services. Even under routine circumstances, cold chain management, vaccine transportation, and human resource constraints can negatively affect vaccination coverage; these challenges might be experienced most acutely in countries with large rural populations.

The findings in this report are subject to at least five limitations. First, UNICEF/WHO coverage estimates are based on the best estimates of a combination of administrative data and survey data, each of which might be subject to overreporting or underreporting. Other factors potentially causing a discrepancy between rotavirus coverage and DTP coverage include the inability to compare the final dose of RV1 to the second dose of DTP, and the lack of data on coverage with the first dose of rotavirus vaccines. Second, although protocols for rotavirus surveillance are standardized across the entire network, there are a limited number of surveillance sites in each country; these might not be representative of pediatric diarrheal illness across the country and might provide an incomplete picture of impact. Third, immunization and surveillance data quality vary among countries. Fourth, not all sites have been able to conduct continuous rotavirus disease surveillance, and data were not included in these results if analysis criteria were not met. Finally, rotavirus surveillance data are not available for all countries before introduction, limiting the ability to assess vaccine impact in countries without vaccine preintroduction data or those that are not part of the ARSN.

Overall, substantial progress has been made in the introduction of rotavirus vaccine and surveillance for rotavirus disease in countries in the WHO African Region. In countries where rotavirus vaccine has been introduced, a substantial decline in the percentage of rotavirus-associated pediatric diarrhea hospitalizations was observed. As rotavirus vaccination coverage increases, an even greater decline might be expected; however, continuous surveillance is a critical component of measuring vaccine impact. Financial support from Gavi, the Vaccine Alliance, has played a key role in rotavirus vaccine introduction and rotavirus surveillance in the region (*10*). Nonetheless, Gavi support will not continue indefinitely; as their economies improve, countries will graduate from Gavi support and begin to finance the total cost of the vaccine. Maintaining surveillance for rotavirus disease will provide important data necessary to promoting continued investment in rotavirus vaccination. Rotavirus vaccination is a critical element in reducing child deaths from diarrhea and contributing to the improvement of child health globally.

**Summary**

**What is already known about this topic?**

Rotavirus is a leading cause of severe pediatric diarrhea worldwide, and a disproportionate number of deaths occur in countries in the World Health Organization (WHO) African Region. WHO recommends rotavirus vaccination for all infants worldwide.

**What is added by this report?**

As of December 2016, 31 of 47 (66%) countries in the WHO African Region had introduced rotavirus vaccination into their national schedules. Among these countries, the overall coverage for the completed series of rotavirus vaccination was 77% in 2016. In 12 countries with available sentinel hospital surveillance data before and after rotavirus vaccine introduction, the proportion of pediatric diarrhea hospitalizations that were rotavirus-positive declined 33%, from 39% to 26%.

**What are the implications for public health practice?**

Continued commitment to improving rotavirus vaccination coverage in the WHO African Region should contribute to reducing the morbidity and mortality associated with this disease. Maintaining and enhancing the existing surveillance network will be critical to the ability to measure vaccine impact.
